# Environmental Criteria in the Spanish Public Works Procurement Process

**DOI:** 10.3390/ijerph14020204

**Published:** 2017-02-18

**Authors:** José Luis Fuentes-Bargues, Mª Carmen González-Cruz, Cristina González-Gaya

**Affiliations:** 1 Departamento de Proyectos de Ingeniería, Universitat Politècnica de València, Camino de Vera s/n, 46022 Valencia, Spain; mcgonzal@dpi.upv.es; 2 Departamento de Ingeniería de Construcción y Fabricación, ETSII, UNED, C/Ciudad Universitaria S/N, 28040 Madrid, Spain; cgonzalez@ind.uned.es

**Keywords:** green public procurement, environmental criteria, tendering, environmental plan, Spanish construction sector

## Abstract

Green Public Procurement (GPP) is defined as a process of contracting products, services, and works with the least possible damage to the environment during their life cycle. In order to improve the knowledge about GPP, a study of the use of environmental tendering criteria in the Spanish public construction sector has been performed. The results of this study show that the use of environmental criteria in Spanish public sector construction procurement is low in comparison to a certain group of countries, known as “Green 7”, in the European Union. Environmental criteria is the fourth criterion in importance, but its weight in the global of the process is much lower than other criteria such as price, memory of the construction process and the delivery time. National administrations use environmental criteria more frequently because they have more resources and staff training about environmental issues. Environmental criteria are more used in the tendering of civil projects and works whose budget exceeds ten million euro due to the environmental impact of these kind and/or size of projects.

## 1. Introduction

Construction activities have a significant impact on the environment [1,2,3], so it is necessary to develop processes that are more friendly to the environment. Some of these tools are the Environmental Impact Assessment process (EIA), Environmental Management Systems (EMS), Sustainable Building Tools (SBT), Eco-labelling and Green Contracts [4,5,6,7,8,9,10].

Public procurement of the construction sector is divided into works, services, and products. According to the regulatory framework of the European Union [11,12], works include buildings, large infrastructures or reforms that result from the construction process. Services define the operations performed by technicians who are involved in the process, such as architects, engineers, cleaning companies or security companies. Finally, products are defined as the different elements or materials used to make the works or to complete the facility.

For works, EIA and SBT are the most used tools. The EIA process is a set of systematic technical studies that provide an estimate of the effects and the importance of a project on the environment [13]. There are a set of projects, as large infrastructures and industrial activities, which are mandatory to be submitted to the EIA process. Buildings or minor facilities are not submitted to EIA and SBT allow one to assess the building sustainability from a list of criteria related to the environment, such as water consumption, energy consumption, thermal or acoustic insulation, etc. Some of these SBT tools are Building Research Establishment Environmental Assessment Method (BREEAM), Leadership in Energy and Environmental Design (LEED), Green Star [8,14] or VERDE, as it is called in Spain [15]. These criteria for assessing the sustainability of buildings could be established from the ISO 21929-1:2011 norm [16]. This standard establishes a core set of indicators to be considered in the assessment of the sustainable development of new or existing buildings, regarding their design, construction, operation, maintenance, refurbishment, and end of life.

For products and for services, such as for example the Ecolabel group of “holiday accommodations” [17], Eco-labelling is the main tool used. It is a voluntary method of environmental performance certification and a form of labelling that is supported by procedures and criteria that are usually defined in standards or regulations [18,19]. Three examples in Europe are Nordic Swan, Blauer Engel, and European Union Eco-label. Furthermore, Eco-labels result from criteria that consider the environmental impacts products (or services) may have throughout their life cycle to ensure that the label gives consumers/users the possibility to choose the products that are least harmful to the environment [20,21]. 

Finally, EMSs are typically used as requirements for services, construction companies or sometimes for the whole construction process [9,22,23]. An EMS is a set of processes and practices that enable an organization to reduce its environmental impact and increase its operating efficiency [18,24]. The EMS itself does not dictate a level of environmental performance that must be achieved; each company’s EMS is tailored to the company's business and goals.

EIA, SBT, Eco-labelling and EMS allow the establishment of positive characteristics for the environment of the infrastructures, buildings, products, or services. These characteristics can be used in the purchasing or contracting process, i.e., can be formulated in Green Contracts. Palmujoki et al. [25] defined Green Contracts as the introduction of one or more environmental aspects in the contracting of products, services, and works. Large and Thomsen [26] defined Green Purchasing as the integration of environmental considerations into purchasing policies, programs, and actions. 

In public procurement, the definitions employed for green contracts are Green Government Procurement (GGP) [27] or Eco-procurement [10], although the more frequently used term is Green Public Procurement (GPP). These terms are defined as “a process whereby public authorities seek to produce goods, services and works with a reduced environmental impact throughout their life cycle when compared to goods, services and works with the same primary function that would otherwise be procured” [28].

Public procurement accounts for approximately 10% to 15% of the gross domestic product of developed countries [29,30], and in other countries, these values are even greater [31,32,33]. In other words, the states themselves and their different organizations are among the primary consumers of products, services, and works in national markets. Public procurement is therefore important for the development of Green Public Procurement and creating a more sustainable construction sector.

The criteria used in contracting (both in the public and private sectors) that account for aspects of the environment are defined as environmental criteria. 

For example, in a work submitted to the EIA process, environmental criteria can be selected from the corrective and/or the compensatory measures proposed in the Environmental Impact Study (EIS) or in the public participation process. Uttam et al. [34] cited air quality improvement, the development of green spaces and reduction of the impact of excavations as some of environmental criteria. Another example of environmental criteria is shown in the work of Parikka-Alhola and Nissinen [35]. In a case study of the purchase of a goods transportation service, they compare three environmental criteria: life cycle assessment, a method for calculating the lifetime cost of emissions and fuel consumption and an environmental criterion that favors new environmentally sound technology.

Analysis of environmental criteria can be considered as a part of the study of GPP, which is an incipient topic of public procurement and sustainable development, so with the objective of enlarge the knowledge of GPP, this research about environmental criteria has been developed. 

The main objective of this study is to analyze the use of environmental criteria in the Spanish public works procurement environment. Furthermore, another objective of this paper is to compare the use of environmental criteria in the Spanish public construction sector with the public construction sectors of other countries. The results contribute to the knowledge about GPP and can help politicians and public contractors know the current situation and to develop more and better the use of environmental criteria. The paper is divided in six sections. The first section is the introduction on the topic. The Background section describes GPP and the regulatory framework in the European Union and Spain. In section three, the method is developed. In section four, the results of the use of environmental criteria in tendering Spanish public works sector are presented, and in section five, the results are compared with those of other countries. Finally, section six presents the conclusions of this article.

## 2. Background

In the World Summit on Sustainable Development (WSSD) held in Johannesburg in 2002, an implementation plan to support regional and national initiatives was created to accelerate the shift towards Sustainable Consumption and Production (SCP) themes and de-link economic growth from environmental degradation. As a result, in June 2003, the Marrakech process was launched as a global multi-stakeholder process to support the implementation of SCP and create the Marrakech task forces with the participation of experts from developing and developed countries to support the implementation of specific projects on specific SCP themes. One of the seven task forces was “Sustainable Buildings and Construction” led by Finland, whose objectives include promoting sustainable public procurement.

In the last decade, GPP has made notable advances worldwide; for example, studies were carried out in the United States [36], South Africa [37,38] and Asia [39,40]. Similarly, ambitious objectives for GPP have been planned in Europe, and a great number of countries have announced their own development programs [27,41]. Therefore, GPP must become one of the primary pillars in the environmental politics of the European Union and its allied members in the near future [42]. Related to this, some research groups have distinguished between “Green 7” (Austria, Denmark, Finland, Germany, Great Britain, The Netherlands, and Sweden) and the “Other 18” [43,44,45] based on the use of GPP. Although development in some countries, the uptake of green procurement is slow. Moreover, innovative solutions are weakly supported by public procurement [46,47].

Some studies have been carried out on GPP dealing with the acquisition of products. Li et al. [48] studied governmental computer purchasing at state level in the USA and determined that environmentally responsible public procurement can also be seen as a driving force in the integration of environmental product policy instruments. Bouwer et al. [43], in their analysis of green criteria carried out from 1000 tender documents in EU countries, showed that among calls for tenders, on average, 45% included some type of environmental criteria, reaching 55% in Finland.

Nissinen et al. [45] studied a sample of calls for tenders from Denmark, Finland, and Sweden between the years 2003 and 2005. They conducted a detailed analysis of the environmental criteria used in a sample of tenders obtained from Tenders Electronic Daily database (TED-database) and noted the difference between “environmental criteria” and “well-defined environmental criteria”. A well-defined environmental criterion is the one for which the purchasing authority has given the information on how a criterion must be fulfilled and verified. Michelsen and De Boer [49] studied the green procurement practices at a local and regional level in Norway. They showed that GPP is significantly more established in large municipalities than in small ones because large municipalities have more resources for establishing a purchasing department which can generate knowledge and develop purchasing strategies.

Parikka-Alhola et al. [44] studied a sample of calls for tenders in Denmark, Finland, and Sweden and found that in the product groups with potential environmental criteria, green criteria were present in 37% of awards decisions. Here, the weight accounted for 5%–20% of the award criteria with an average weight for green criteria of 3.3%. 

In their econometric analysis, Testa et al. [50] showed the dimension of public authorities and that the level of awareness of the existing tools for supporting GPP have a positive and significant effect on the probability that they adopt GPP practices. Igarashi et al. [51], in their analysis of a sample of Information and Communication Technology (ICT) tenders from Norway, show that the environmental criteria were the third most frequent award criteria after price and quality, but the average weight was lower than all other award criteria. 

Testa et al. [52] analyse some aspects of GPP through a survey of managers in charge of administrative functions at the municipalities of Tuscany (Italy), and they show that it is necessary to develop successful strategies, well-trained personnel and dispose of guidelines and tools for GPP. 

Most of the research on GPP is based on questionnaires filled in by the contracting authorities and the purchasing departments of private companies (analysis of environmental criteria in the private sector). One of the common conclusions is that the respondents tend to exaggerate the application of environmental criteria or practices, which are unrealistic when contrasted with the calls for tenders and the subsequent contractual clauses [25,44,50,53,54].

An additional difficulty identified in all GPP studies is the vagueness and lack of clarity of the environmental criteria themselves [33,43,49]. In many occasions, highly generic criteria are used, making it difficult for the contracting authority to verify that they have complied with the criteria. In fact, many times, contracting authorities do not consider this option because the contracts do not include the monitoring and inspection of the environmental conditions [25].

Research into works and services has not been given sufficient attention [54,55,56], especially in the definition and application of the criteria in the environmental construction sector. Lam et al. [9] concluded that construction companies in China with EMS have similar attitudes towards green specification as companies without EMS and that simply promoting EMS in the construction industry is not sufficient to force the inclusion of green considerations in construction procurement. 

### Regulatory Framework in the European Union and Spain

The award of a contract by an administration depends on a number of endpoints. In the European Union and in Spain, Directive 2004/18/EC [57] and Royal Decree 3/2011 [58] respectively, regulate public procurement and describe the tendering criteria that allow the contracting authority to select the Economically Most Advantageous Tender (EMAT). These criteria include price, quality technical merit, aesthetics and functional characteristics, environmental characteristics, running costs, profitability, customer service, technical assistance, delivery date and execution time.

The EMAT, based on several criteria, is traditionally called the procedure contest. The bid is based solely on a single criterion, which inevitably is the price, and the procedure is traditionally known as auction. 

The evaluation criteria used can be divided into two groups: the criteria evaluated by formulae and those evaluated by value judgments. For the former, various predetermined formulae can be employed, including aspects such as price, delivery time, and the necessary labour for the project. However, the scores for the criteria assessed by value judgments will always contain some subjective bias by the individual who performs the evaluation. Environmental characteristics can be assessed by value judgements or by formulae, as when data are available from a Life Cycle Analysis (LCA).

The environmental criteria are regarded as a necessary justification that relates the stipulated requirements to their importance from an environmentally friendly point of view [25]. The European Supreme Tribunal of Justice (ESTJ) has ruled that environmental criteria, emissions and noise, for example, must be clearly specified and measurable. This means general and unmeasurable environmental criteria need not be considered. This aims to make the tendering process transparent and equal for everybody [25]. In its 1998 report [59], the European Commission indicated that introducing the environmental aspect into tendering should involve financial advantages for public administrations. The ESTJ established that the environmental criteria did not necessarily have to include an economic dimension [60].

In 2003, the European Commission Report COM 2003/302 [61], marked as one of the targets for environmental improvement, stated that both public and private consumers should be in possession of the maximum information available when selecting a product. One of the means of achieving this objective in the public sector was introducing environmental considerations into public procurement.

The possibility of using environmental aspects in Spanish public procurement already existed before the creation of European Directive 2004/18/CE. In the third ruling of Law 11/1997 dealing with Containers and Container Residue [62], public organizations were obliged to promote the use of reusable and recyclable materials in contracting public works and provisions. As a consequence of this law, the Ministry of Environment issued an Order on 14 October 1997, specifying the criteria to be included in the environmental evaluations, in the particular administrative tenders applicable in contracts drawn up by the Ministry of Environment [63]. For the other Spanish administrations, the Law 48/1999 introduced environmental criteria into tenders in Spain, modifying their initial interpretation in accordance with the ESTJ ruling.

One of the criteria used in the Spanish public procurement was the ISO 14000 Environmental Management System Certification, but such the Advisory Board on State Administrative Contracting (ABSAC) stated, it is a criterion of technical solvency and not as an objective criterion for the adjudication of the contract [64].

In the European Union, parallel regulatory actions have been enacted to promote the acquisition of environmentally friendly products, including Directive 2002/91/CE [65] and Directive 2010/31/UE [66], obliging all buildings, for both residential uses and others, to fulfil the energy efficiency requirements. This European directive has also been adopted in Spain in the Royal Decree 235/2013 [67], which stipulates the obligatory nature of an energy efficiency certificate in all buildings for sale or rent, providing information on the energy characteristics of the property.

Another action of the European Union, for both the building and civil engineering subsectors, is the promotion of the use of Eco-labels and life cycle analysis for the acquisition of products [68,69]. Four large groups are proposed as possible environmental criteria for awarding works in the building subsector. The first is energy efficiency for both the building as a whole, as well as the equipment installed. The second aspect involves construction materials. The demand for sustainable building supplies and the use of life cycle analysis as a tool for selecting efficient materials with a long life cycle are suggested. The third and fourth aspects are measures designed to facilitate waste management (reduction, reuse, and recycling) and the responsible use of water (for example, more rational usage or use of rainwater and grey water).

In order to verify the application of some of these measures, a review of the academic literature about public procurement in Spain was performed. Studies have been focused on the selection of contractors and tendering criteria, not in green public procurement. Ballesteros et al. [70,71] have developed prediction models based on historic time series of auctions and tenders. Pastor et al. [72] focuses on the application of multi-criteria Analytic Hierarchy Process (AHP)-Analytic Network Process (ANP) methodologies or the selection of criteria in public contracts. Bendaña et al. [73] studied the implementation of neural networks and diffused techniques in the selection of contractors. Fuentes et al. [74,75] have developed some studies about the Abnormally Low Tenders (ALT), the Economic Scoring Formulae (ESF) and the current status of the Spanish public procurement. Bendaña et al. and Pastor et al. proposed different environmental criteria in their methodologies, such as the environmental characteristics of the company, the environmental management system of the company, and the environmental control of the project. In the development of methodologies with the objective of facilitating their application, the number of criteria is reduced, incorporating the environmental criteria into the criteria corresponding to the project study.

The analysis of the Spanish laws and literature review show environmental criteria can be used in the public procurement for a long time but there is not a study about its use and its importance, so the present research can be added to the body of knowledge, both at national and global level.

## 3. Methods

The method used in this study is a content analysis of the documents obtained from calls for tenders. Some researchers have used this method in order to study the use of GPP [25,44].

A sample of one hundred cases of public procurement of works between the years 2008–2011 was obtained from the contracting authorities. The data used in this study were projects and tendering documents and they were obtained from the web pages of the contracting authorities and from national databases. This process began in 2011 and continued for a period of six months. 

**Figure 1:**
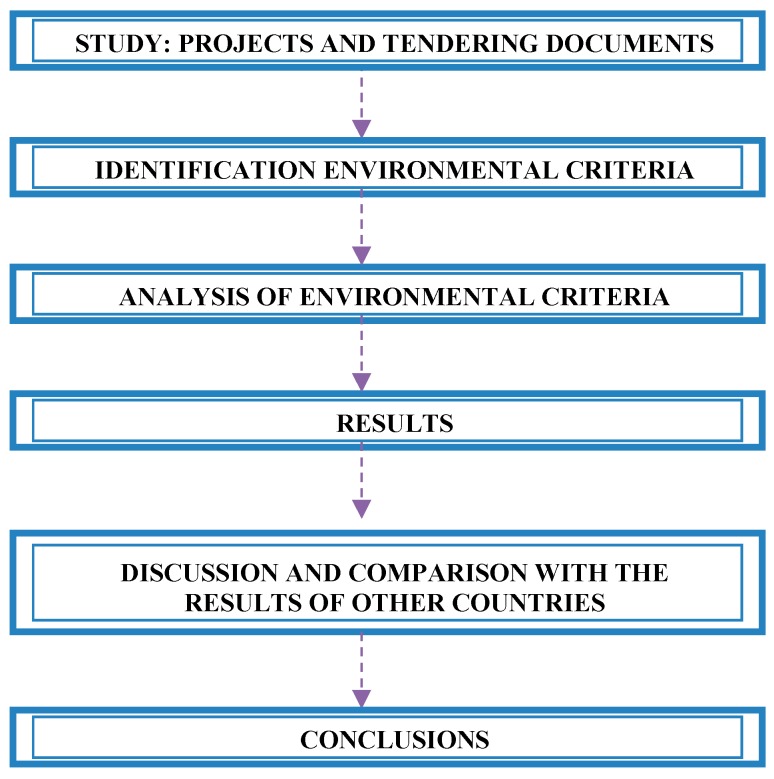
Study method steps.

The method is divided into six steps (Figure 1). The first step is to study the project and the tendering documents. In the step two, each case of the sample was analysed to locate any environmental criteria involved in the tenders. These environmental criteria are analysed and classified by subsector (civil engineering or building), geographical scope and project budget. Thereafter, the weight of the environmental criteria is analysed and classified by subsector, geographical scope, and contract execution budget. Finally, the environmental criteria identified are related with other criteria used in the tendering process. In the next step, a discussion and comparison with the results from other countries is included and some measures are proposed to improve the use of environmental criteria in Spanish tendering processes. Lastly, the conclusions are presented.

This analytical method could serve as a guide for future work in order to compare results from the use of other criteria in public procurement, such as social criteria, or to compare the results with the use of environmental criteria in the procurement of products and services. The limitation of this method is the difficulty of getting comprehensive information on the projects, as only part of the documentation is available to bidders and the public.

The files were classified according to various criteria. One of these was the period in which the tendering process took place, with 37% tendered in 2010, 52% in 2011, and 11% in other years. 

The construction sector is divided into two subsectors. The building subsector includes all types of buildings: housing, factories, offices, schools, and sports facilities. Civil engineering work includes roads, ports, airports, railways, and water supply pipelines, among others. The sample was composed by 47% of public works and 53% of buildings.

The Spanish administration structure is divided into national scope (central government with ministries and public enterprises), autonomous scope (17 autonomous communities and two autonomous cities (Ceuta and Melilla)), provincial scope, and local scope. Each autonomous community is divided into provinces (provincial scope); e.g., Madrid has one, and Castilla-León has nine. The provinces include cities, towns, and villages with their own local administrations acting at the local scope.

Local administrations composed 42% of the sample (Provincial 5%, Autonomous 37% and National 16%). The territorial distribution of the works in the sample includes at least one tendering process in each of the Spanish autonomous communities. The sample was composed by 93% of contests and 7% of auctions.

Four price levels were established according to the execution budget of the projects. The four levels were between €200,000 and €1,000,000 (23%), between €1,000,001 and €5,000,000 (44%), between €5,000,001 and €10,000,000 (22%) and over €10,000,001 (11%). 

## 4. Results

The results obtained in the tendering analysis show that 35% of the projects studied include references to environmental criteria. The definitions and descriptions of the different environmental tendering criteria identified in the study sample are described in Table 1.

As can be seen in Table 1, the possession of Eco-Management and Audit Scheme (EMAS), ISO 14001, or similar certificates does not appear as an environmental tendering criterion, so the rules of the European Union and Spanish laws are fulfilled.

All the environmental criteria described at Table 1 are valued judgements criteria, there is not criteria valued through formulae, and so environmental criteria can be considered for this sample as a value judgement criteria. Also in the tendering documents there are not a list of minimum requirements for the environmental criteria neither partial scores in function of some of the items offered.

The description of the different environmental criteria is quite similar, it is a list of environmental matters that must be taken into account or a series of possible measures (energy saving, the use of renewable energy, the use of sustainable materials with a long life cycle, etc.) to avoid the degradation of the environmental related with the work. These measures may be found in the GPP recommendations from the European Commission for the contracting of construction works and civil engineering [68].

The distribution of environmental criteria in the construction sector shows that 22 of the 35 tenders belong to the public works subsector (62.9%) and 13 belong to the building subsector (37.1%). In the former subsector, the tenders with environmental criteria account for 22 out of 47 total tenders, i.e., 46.8%. In the building subsector 13 out of 53 tenders had environmental criteria, i.e., 24.5%, which shows that the use of environmental criteria is more common in civil engineering projects.

According to geographical scope, the results show that environmental criteria are used mostly widely by the administrative bodies at the national level, in 62.5% of the cases (10 of 16 projects). In the autonomous regions, this figure was 29.7% (11 of 37 projects), and for the local administrations, 33.3% (14 of 42 projects). There were no cases of environmental criteria being used at the provincial level. 

In the analysis between tenders with environmental criteria and the Contract Execution Budget (CEB), the results show that 36.4% of the projects with a CEB between €1,000,001–€5,000,000 and 36.4% of the projects with a CEB between €5,000,001 and €10,000,000 contain environmental criteria. At a national level, 72.7% of the tenders with a CEB greater than €10,000,000 have environmental criteria and only 13.1% in projects with a CEB lower than €1,000,000.

The weighting of the environmental criteria in the total tender was studied. The maximum weight of the environmental criterion is 15 points of a total of 100 points. The average weight of the 35 works of the sample is 5.7 points over a hundred. The most used weight is “5 points”, occurring on 10 occasions.

**Figure 2:**
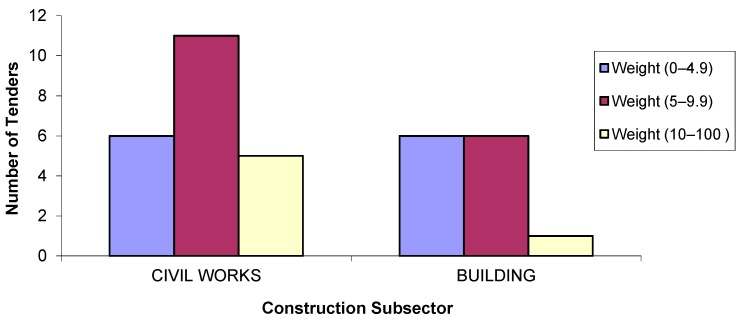
Number of tenders according to weighting of environmental criteria by the construction subsectors.

In Figure 2, the weighting of the environmental criteria is related to the subsector. The environmental criterion has more weight in civil engineering projects than in building projects, in which the weighting was above 10 points in only one of 13 cases.

**Figure 3:**
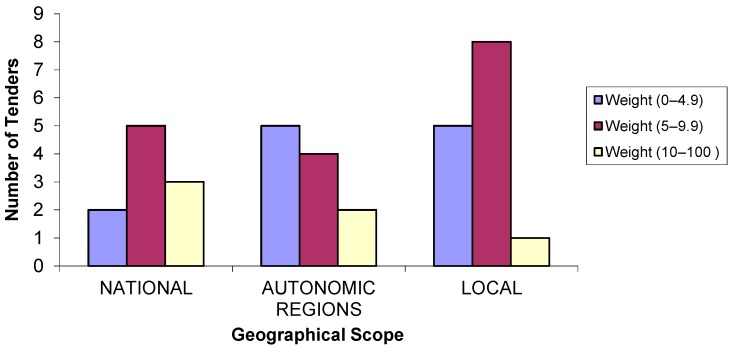
Number of tenders according to environmental criteria weighting by the geographical scope of the administration.

In Figure 3, the distribution of the weighting of the environmental criteria is related to the geographical scope of the administration. In 57.1% of the tenders of local administrations, the weighting of the environmental criteria ranged between 5 and 9.9 points out of 100. In the autonomous regions, five projects were between 0 and 4.9 points out of 100, and four projects were between 5 and 9.9 points out of 100. At a national level, 50% of the tenders were weighted between 5 and 9.9 points.

**Figure 4:**
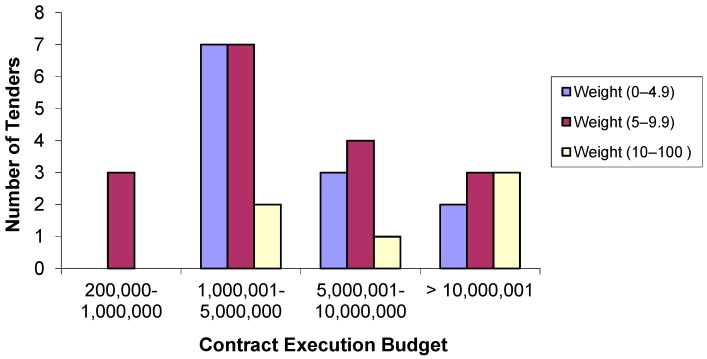
Number of tenders according to environmental criteria weighting by contract execution budget (in euros).

If a comparison is made between the weight of the environmental criteria and the CEB (Figure 4), the results show that the most used weighting range for environmental criteria is between 5 and 9.9 points, with 100% for projects with a CEB between €200,000–€1,000,000, 43.8% for projects with a CEB between €1,000,001–€5,000,000, 50% for projects with a CEB between €5,000,001–€10,000,000 and 37.5% for projects with a CEB greater than €10,000,000.

If the sample study is analysed regarding all variables but with the geographical area as a starting point, the following points can be noted:

- National: There are no tenders in the sample of the building or public works subsector lower than €1,000,000. The maximum weight of the sample (15 points) is for a public works project for more than €10,000,000. The most frequent weighting is 7.5 points (out of 100), and the average weighting is 6.4 points.
- Autonomous Regions: There are no tenders worth more than €10,000,000. Works with budgets lower than €1,000,000 are public works with a weighting between 5 and 9.9 points. The maximum weighting is 10 points (used twice), and the most frequent weighting is 5 points (four times). The average weighting is 5.1 points.
- Provincial: None of the tenders in this geographical area involved environmental criteria.
- Local: The distribution of tenders is more dispersed in this sector. The maximum weighting of the environmental criterion is 10 points, the most frequent is 5 points (five times), and the average weighting is 4.7 points. The tender with the lowest weighting (2.5 points) in the sample is for a public works project with a budget of over €10,000,000.

Another aspect studied was the relationship between environmental criteria and other criteria used in the tendering process. The main criteria have been identified in the 35 projects from the sample. These are, in order by number of appearances, the price (all projects), a descriptive memory of the construction process (33 of 35 projects), the delivery time (33 of 35 projects), the quality systems (24 of 35 projects), the health and safety facilities and procedures (21 of 35 projects), the technical team (14 of 35 projects) and the improvements (11 of 35 projects). No relationship can be established between the weight of the environmental criterion (5.7%) and the weight of the other criteria, but the relative importance of the weight of each criterion in the study sample has been noted. The price is the criterion with the highest weight (47.6%), followed by the memory of the construction process (15.7%) and the delivery time (9.3%). Some other criteria have similar weights to the environmental criteria, such as improvements (4.5%), quality systems (3.8%) and health and safety facilities and procedures (3.3%).

As a final point, a noteworthy characteristic in the study sample is the system of awarding university contracts. Environmental criteria appeared in eight of the 12 cases studied (66.7%), in seven of which the work was in the Building subsector, with an environmental criterion weighting between 3 and 5 points on every occasion. The project involving the civil engineering sector (a work promoted by the University of Valencia) had a weighting for the environmental criteria of 7.5 points. 

## 5. Discussion

### 5.1. Comparison with the “Green 7”

In the study sample, 35% of the Spanish public administrations use environmental criteria when contracting construction projects. This could be considered as a respectable quantity, but if this value is compared with studies conducted between 2005 and 2006 in the so-called “Green 7” countries it is actually a very low value, especially when taking into account the difference of five years. For example, in the study of 2005, environmental criteria was used more 80% for Sweden, between 70%–80% for Germany, between 60%–70% for Austria, Denmark and United Kingdom and between 50%–60% for Netherlands and Finland [43,44,45]. In comparison with a study conducted in Italy in 2010 by Testa et al. [50] the results are very similar (35% include environmental criteria in the technical evaluation of the companies competing for the tender and 23% as tendering criteria). These two examples confirm the division between the “Green 7” and the “Other 18” made by Bouwer et al. [43] in the study on GPP in the European Union.

### 5.2. Description of the Environmental Criteria

Table 1 shows the description of the environmental criteria used at the contracts of the sample. All the criteria are assessed as a value judgement and this is all the information for bidders to prepare the technical document of the bid and also is all the information for the administration’s technical staff to assess the bid. 

For example, in various criteria, appears the descriptor “Analysis of the organization”. Bidders can suppose that it is the environmental organization of the company or can also suppose that is the environmental organization of the technical staff of the work. Other example could be the descriptor “Reduction of noise levels”, it can mean only a description of actions aimed at the reduction of the noise levels or can mean a description of actions with the numerical reduction of the parameter noise.

These are two examples of descriptors included in many of the environmental criteria pointed in the research, so it is necessary a better definition of the environmental criteria by contract authorities.

### 5.3. Environmental Plan

The most frequent environmental criterion found in this study is the drawing up of an Environmental Plan (EP). As can be seen in description of the environmental criteria (Table 1) there is a similarity in many of the descriptors of the criteria, so it is necessary a standardization and should be developed a guide for the EP. The information for drawing up the EP must be obtained from the Project, the Environmental Impact Statement (or similar document), the Record of Decision (in the event that the project is subject to an Environmental Impact Assessment), environmental politics, the environmental procedures of the tendering company, and the specific conditions laid down in the contracting of the work. Uttam et al. [34] justified the relationship between EIAs and GPP and the necessity of developing a clear and systematic link between EIA and GPP in order to incorporate the conclusions of the EIA as environmental criteria in the procurement. 

The EP should not be merely considered as a document for the evaluation of the environmental criterion during tendering. It should be regarded as a binding document on environmental matters between the administration and the contracting company once the project has been awarded. This would allow the Project Manager and the Environmental Project Manager (if there is one) [76,77] to track and control the environmental measures and actions. The EP should be kept active during the execution of the work and it should adapt to the management systems and environmental measures initially proposed to the different situations and the needs that occur during the process.

### 5.4. Environmental Management Systems

EMS, ISO 14001, or similar tools are essential for promoting good environmental practices in companies and achieving a sustainable construction system. However, their use as criteria for awarding contracts is controversial. Still, they could act as a filter, which goes against the principle of equality, a fundamental pillar of European public procurement [57]. Environmental criteria must allow for choosing the best offers in relation to aspects directly associated with the contract. The environmental management systems emphasize more indirect or complementary aspects [9,50]. In this case, rules from European directives are fulfilled, and EMS are not used as criteria.

### 5.5. Green Criteria Across Different Types of Works

The use of environmental criteria is more common in procuring civil engineering works. There are few studies that note this subdivision, but the one carried out by Varnäs et al. [54] on GPP in the Swedish construction sector shows a similar trend.

The dimension of public administration has a certain influence on the use of environmental criteria [49,50]. The environmental criteria can be associated with the geographical scope study. National administrations use this type of criteria more than provincial or local administrations. Among the causes that explain this behaviour are the following: the lack of technical means and environmental knowledge necessary to prepare the contract documents in small administrations, the non-existence of technical guides (prepared by the larger administrations) to facilitate the use of environmental aspects in contracting, and lastly, the technical difficulties that might arise during the subsequent evaluation of tenders [33,43,47,49,52,78].

### 5.6. Comparing Environmental Weights with Other Studies

The average weighting of the environmental criterion in this study compared to the total tender is 5.7 points out of 100, slightly higher than the 3–5 points obtained by Palmujoki et al. [25] in their research in Sweden and Finland and the 3.3 points obtained by Nissinen et al. [45] in Sweden, Finland and Denmark. This weight is in the fork of the results obtained by Igarashi et al. [51] in Norway. Environmental criterion is the fourth criterion in importance in the study sample (5.7% of weight), after price, memory of the construction process and the delivery time. These results can be compared with the results obtained by Igarashi et al. [51], where environmental criterion was third in frequency (after price and quality) but sixth in weight, after price, quality, delivery time, service and maintenance and cost. According to the Igarashi et al. results and the results from the study could be affirmed that the importance of environmental criteria, both products and works, is still low and GPP has a long way to be implemented at public procurement. 

In the same way, the low weights of environmental criteria in comparison with other criteria obtained in this study and in the study of Palmujoki et al. [25] allow to affirm that bidders can conceive environmental criteria as secondary obligations in the bidding process and often makes their study or analysis irrelevant in achieving the work contract. 

The results of the study show how the use of environmental criteria is more widespread in projects with budgets higher than €10,000,000 (72.7%) and practically residual in those with budgets below €1,000,000 (13.1%). The financial approach tends to have a more superior weight in the lower-priced tenders than in those with higher amounts [79]. This appears to show a certain relationship between the project’s budget and the use of other tendering criteria apart from price, for instance, environmental criteria.

## 6. Conclusions

The results of this study show the use of environmental criteria in Spanish public sector construction procurement is low (35%) in comparison to a certain group of countries, known as the “Green 7”, in the European Union. Spanish authorities must make greater use of environmental criteria and one of the first steps would be to develop plans for environmental training of the administration’s technical staff.

The use of environmental criteria in the tenders is higher at national administrations than local or provincial administrations and at projects with higher budgets (in this case more than €10,000,000). This is because national administrations have more economical and technical resources, and their staff are more highly trained in environmental matters for assessing the bids.

The average weighting of the environmental criteria was found to be low (5.7 points out of 100), the fourth in importance in the study of the sample after price, memory of the construction process and the delivery time, but with much difference respect to the previous three. It seems that environmental criteria are therefore, secondary for public authorities and bidders. 

Environmental criteria are often not very well defined by administrations in the tender documents and usually they are assessed as a value judgement, making it difficult for bidders to prepare their bids and for the administration’s technical staff to assess them. One of the aspects that should develop is the use of the EP as environmental criterion. A content guide of the EP must be defined and depends on the type of work, it would be adapted and it would reflect the measures to protect the environment to be implanted by the organization during the execution of a project. The EP should be considered also as a contractual document on environmental matters between the administrative body and the contractor. 

GPP is an important topic that should be further developed in future because results of the research could help public authorities, project managers, bidders and many stakeholders related with the public construction sector to improve the practical application of measures which promote a sustainable development. With the method detailed in this paper, and given that each administration is free to select its own tendering criteria, a study about the use of environmental criteria in each Spanish autonomous community could complement this research. 

Other aspect which should be worked in the future is the development of environmental criteria which quantify the proposed measures of the bidders and can be assessed through formulae in the tendering process. 

## Figures and Tables

**Table 1:** Environmental Criteria and its description in the study sample.

Description of the Environmental Criterion	Number of Tenders	Description
Environmental Action Plan	7	Environmental organization chart of the work team Environmental management measures: management of land and building materials, country restoration, reduction of waste generation, reduction of air pollution, reduction of water pollution, reduction of soil pollution, noise reduction, reduction of visual impact, reduction of impact on fauna and flora, reduction of use of fossil fuels and use of renewable energy Proposals of materials to be used: recycled, reusable, etc.
Environmental Action	5	In the execution activities of the works: Use of appropriate environmental materials and products Minimize waste production and recycling Limit or, where appropriate, avoid water consumption Minimize energy consumption, prioritizing the use of alternative energies Minimize noise pollution Location of construction sites, where environmental impact will be minimized Use of environmentally appropriate machinery and equipment
Environmental Measures	4	Reduction of noise levels caused by equipment and machinery Reduction of the emission of gases Reduction of waste generation Use of eco-labeled products or other equivalent quality marks Reduction of fossil fuel use Use of alternative energies or energy saving mechanisms Use of recyclable, reusable or recoverable materials Use of materials from recycling processes Use of materials that reduce the noise emission levels
Improvements in Environmental Matters	3	Reduction of noise levels caused by equipment and machinery Reduction of the emission of gases Reduction of waste generation Use of eco-labeled products or other equivalent quality marks Reduction of fossil fuel use Use of alternative energies or energy saving mechanisms Use of recyclable, reusable or recoverable materials Use of materials from recycling processes Use of materials that reduce the noise emission levels
Management of Paperwork and Execution of Environmental Measures	2	Reduction of waste generation Reduction of noise levels Reduction of the landscape damage Reduction the risk fire Measures proposed to minimize the effect on natural vegetation Measures proposed to minimize the temporary occupation during the execution of the works Improve proposals to ensure the correct integration of works
Environmental Activities and Sustainability Criteria	2	No description
Quality and the Environment	2	Environmental measures according to the object of the contract
Environmental Quality	2	Use of recyclable, reusable or recoverable materials Preventive, corrective and compensatory measures according to the Record of Decision or/and the Environmental Impact Study Certification of timber and/or forestry products derived from sustainably managed forests
Environmental Management Plan for the execution of the work	2	Analysis of the organization, equipment and systems of the company to avoid environmental impacts in the execution of the works. Environmental measures for saving energy, measures to reduce the emitted radiation of the systems and the equipment and measures to reduce the noise pollution
Research and development of environmental measures	1	No description
Relationship of environmental measures proposed for adoption during the execution of the works	1	Reduction of noise levels Reduction of the emission of gases Reduction of waste generation Management Plan of waste Use of recyclable, reusable or recoverable materials Use of alternative energies
Environmental Security, Health, and Actions	1	Environmental Action Plan: identification of units of work which could generate environmental impacts and corrective measures
Quality Management, Prevention and Environment, and Research & Development & Innovation (R&D&I)	1	No description
Management and Execution of the contract with procedures that promote quality and respect for the environment.	1	No description
Investment in Environmental Program	1	No description
